# Arsenic Speciation and Extraction and the Significance of Biodegradable Acid on Arsenic Removal—An Approach for Remediation of Arsenic-Contaminated Soil

**DOI:** 10.3390/ijerph14090990

**Published:** 2017-08-31

**Authors:** Thinh Nguyen Van, Yasuhito Osanai, Hai Do Nguyen, Kiyoshi Kurosawa

**Affiliations:** 1 Graduate School of Integrated Sciences for Global Society, Kyushu University, Fukuoka 819-0395, Japan; osanai@scs.kyushu-u.ac.jp; 2 Soil Science Department, Faculty of Land Management, Vietnam National University of Agriculture, Hanoi 100-000, Vietnam; haisardc@gmail.com; 3 Institute of Tropical Agriculture, Kyushu University, Fukuoka 812-8581, Japan; kurosawa@agr.kyushu-u.ac.jp

**Keywords:** arsenic contamination, sequential extraction, biodegradable organic acid, soil washing, soil remediation

## Abstract

A series of arsenic remediation tests were conducted using a washing method with biodegradable organic acids, including oxalic, citric and ascorbic acids. Approximately 80% of the arsenic in one sample was removed under the effect of the ascorbic and oxalic acid combination, which was roughly twice higher than the effectiveness of the ascorbic and citric acid combination under the same conditions. The soils treated using biodegradable acids had low remaining concentrations of arsenic that are primarily contained in the crystalline iron oxides and organic matter fractions. The close correlation between extracted arsenic and extracted iron/aluminum suggested that arsenic was removed via the dissolution of Fe/Al oxides in soils. The fractionation of arsenic in four contaminated soils was investigated using a modified sequential extraction method. Regarding fractionation, we found that most of the soil contained high proportions of arsenic (As) in exchangeable fractions with phosphorus, amorphous oxides, and crystalline iron oxides, while a small amount of the arsenic fraction was organic matter-bound. This study indicated that biodegradable organic acids can be considered as a means for arsenic-contaminated soil remediation.

## 1. Introduction

Arsenic (As) is a toxic element that ranks 20th in abundance in the Earth’s crust, with an average concentration of 2–3 mg/kg [1,2]. Arsenic is ubiquitous in the atmosphere, lithosphere, hydrosphere, pedosphere and biosphere of the Earth and is commonly associated with metal ores, such as arsenolite (As_2_O_3_), olivinite (Cu_2_OHAsO_4_), cobaltite (CoAsS), and proustite (Ag_3_AsS_3_) [3]. High concentrations of arsenic in groundwater (over 10 µg/L) can adversely affect human health [4,5]. Exposure to large amounts of inorganic arsenic in the soil, sediment and surface water has proven to be harmful to organisms [6,7,8,9].

Through the weathering process, arsenic from both natural and anthropogenic sources can be transported and accumulated in the soil, groundwater, sea water, sediment, and human food chain [10,11]. Recently, many studies have reported that soils and sediments in estuarine areas, which are primarily used for aquaculture production, are contaminated by arsenic [12,13,14,15]. Arsenic is also accumulated in plants and aquaculture products, such as mangroves, fish, and oysters [14,16,17,18,19,20,21]. However, the toxicity of arsenic depends on the oxidation state, chemical species and bioavailable dose in the environment [2,22]. Thus, a risk assessment that includes information on the potential mobilization of As in soils and sediment is necessary.

One method for determining the relative lability of arsenic in soils is a sequential extraction with reagents of increasing dissolution strength [23]. These operationally-defined forms can help to estimate the amount of arsenic in different fractions that could be mobilized due to changes in the soil media [24]. In addition, based on the number of arsenic fractions, we can propose a suitable procedure for arsenic-contaminated soil remediation that focuses on the targeted forms of arsenic, especially for the soil washing method.

During the past decades, several studies have been conducted to evaluate the effectiveness of various single chemical agents on arsenic removal in soils and sediments, such as HCl acid, H_3_PO_4_ acid, ethylenediaminetetraacetic acid (EDTA), Na_2_EDTA, and surfactants [25,26,27,28]. Some biodegradable organic acids (e.g., oxalic acid, citric acid, and ascorbic acid), which are active components in the root exudates of plants [29,30], were also reported to have a significant effect on arsenic extraction from soils and sediments [31,32,33]. These acids attain much higher anionic metal extractions compared to other chelate agents, such as EDTA or Na_2_EDTA [34,35,36]. However, most of the studies have focused on a single agent, and the factors and conditions affecting the extraction process of estuarine soils in Vietnam are still unclear. Therefore, there is a need to optimize soil washing agents as well as their concentrations. For these purposes, four contaminated soil samples from different land uses in the Ba-Lat estuary, Red River Delta, northern Vietnam, were used in the present study. In the first part of this study, the sequential extraction of arsenic was applied by modifying the protocol of Wenzel et al. [23] taking into account the anionic species of arsenic in soil. In the second part, the optimal washing conditions for soil treatment with single and multiple washing agents that consisted of citric acid, oxalic acid and ascorbic acid were investigated. In the third part, the experiment on washing effectiveness for arsenic removal was assessed based on the arsenic fractions of treated soil samples from the previous part.

## 2. Materials and Methods

### 2.1. The Soil Samples

A total of four contaminated soil samples were used for the experiments in this study. These samples were collected from four sites (Figure S1), which are located in different land uses in the Ba-Lat estuary (BLE), including river bed (R), mangrove forest (C), paddy field (T) and an extensive aquaculture farm (S). The total concentration and spatial distribution of arsenic in the BLE area, which is the largest estuary of the Red River in northern Vietnam, were documented by Thinh et al. [12]. At each sample site, top layer (0–10 cm) soils were collected from five spots in a 10 m^2^ area. After collection, the soil sample was dried and sieved through a 2-mm stainless steel sieve. Five soil samples from each site were mixed homogeneously and stored in polyethylene bags for further use.

### 2.2. Experimental Design

#### 2.2.1. Sequential Extraction Protocol

A modified sequential extraction scheme for arsenic was developed by combining the fractions reported in the existing methods [23,24,37,38,39], as described in Table 1. In the sequential extraction, the soil samples were treated with different extraction solutions and reaction conditions in a sequence to collect the individual fractions from each extractant for As analysis. Six fractions were chosen to define the partitions of arsenic in soils, including non-specifically adsorbed As (F1); specifically adsorbed As (F2); Arsenic associated with amorphous hydrous Al-Fe-Mn oxides (F3); Arsenic associated with crystalline Fe oxide (F4); Arsenic bound to organic matter (F5); and a residual fraction (F6).

To minimize solid material losses, one gram of soil was placed in 50 mL centrifuge tubes with 25 mL of the selected extraction solutions, except for the F5 extraction. After the extraction step, the tubes containing the soil and the extractant were centrifuged for 15 min at 10,000 rpm. The supernatant was removed and filtered through a 0.45 µm cellulose acetate filter in polyethylene bottles, and As concentrations were determined following the description below. Between each step, 10 mL of deionized water was used to wash the residue. After centrifugation for 15 min, the supernatant was discarded. This step was repeated three times.

#### 2.2.2. Washing Experiments

For each sample, ten treatments were performed to observe the release of arsenic using two single organic acid solutions, including oxalic and citric, and two acid mixtures that consisted of oxalic-ascorbic acid solution and citric-ascorbic acid solution. The concentration of the single acid solutions contained 0.01 M, 0.05 M, 0.1 M and 0.2 M, while the organic acid mixture solutions contained 0.2 M oxalic acid (or 0.2 M citric acid) and 0.1 M ascorbic acid. All organic acid solutions, including single acid solutions and combined acid solutions, had a pH value of 3.5 that were adjusted using 0.1 N HCl and 0.05 N NaOH. The suspensions, which had 1 g of soil and 20 mL of selective acid solution, were shaken in polyethylene centrifuged tubes at room temperature (25 °C) for 24 h at 155 rpm. After shaking, the supernatants were removed using the same protocol as in the sequential extraction. After the washing step using a combination of 0.2 M oxalic + 0.1 M ascorbic, and 0.2 M citric + 0.1 M ascorbic, the arsenic fractions in the soils were analyzed using the sequential extraction protocol as described in Section 2.2.1.

### 2.3. Analytical Methods and Statistical Analysis

Approximately 0.5 g of soil, which was processed as stated above, was used for digestion using the United States Environmental Protection Agency 3050B method [40] to obtain the total concentration of arsenic. Each soil sample was digested and analyzed in triplicate.

The soil digest solution as well as the extracted solutions in the washing experiments were analyzed for arsenic and aluminum concentrations using inductively coupled plasma-mass spectrometry (ICP-MS, 7500ce, Agilent Technologies, Inc., Santa Clara, CA, USA), while iron was measured using atomic absorption spectroscopy (AAS, Hitachi Z-2300, Hitachi Science & Technology, Tokyo, Japan).

The organic carbon content (OC) in the soil was measured using the Walkley method [41]. The cation exchange capacity (CEC) of the soil was determined by the ammonium acetate method [39]. The content of the soil particle sizes was analyzed using the pipette method [42]. The pH was measured using a Horiba pH meter (D55, Horiba Ltd, Kyoto, Japan), in which the ratio of water to the soil was 5:1 [43].

All reagents were of analytical grade, and double-deionized water (Milli-Q Millipore 18.2 MΩ/cm) was used for dilutions. All extractions were conducted in duplicate. The extracted solution, which could not be immediately measured, was digested by nitric acid and stored in the freezer at 4 °C.

Statistical analysis and data visualization were performed using R version 3.3.1 [44] in R-studio version 0.99.902 [45].

## 3. Results

### 3.1. Soil Characterizations

Prior to the washing experiments, the soils were characterized, and the mean values of major chemical properties as well as the coordinates of the soil samples are given in Table 2. The mean pH values of the soil samples were near neutral and ranged from 6.52 to 6.98. Total organic carbon content was low with a range of 0.7% to 1.5%. The cation exchange capacity (CEC) was slightly variable with a range from 11.0 cmol kg^−1^ in the mangrove forest soil sample to 16.0 cmol kg^−1^ in the aquaculture farm sediment sample. The particle size distribution of four soil samples was highly variable, especially in the clay and sand contents. River bed sediment had the lowest content of clay (5.5%) compared to the paddy soil and aquaculture sediment with clay contents of 25.3% and 26.2%, respectively. As shown in Table 1, the silt was dominant in four soil samples with a range from 44.4% in aquaculture sediment to 58.1% in paddy soil.

Total arsenic concentrations for all samples decreased in the order of River bed (R) > Mangrove forest soil (C) > Aquaculture farm sediment (S) > Paddy soil (T), with a range from 16.84 µg/g to 31.00 µg/g. Concentrations of arsenic in all soil samples exceeded 12 µg/g, which is the permissible level for agricultural soil in Vietnam [46].

### 3.2. Sequential Extraction of Arsenic in Soil

**Figure 1:**
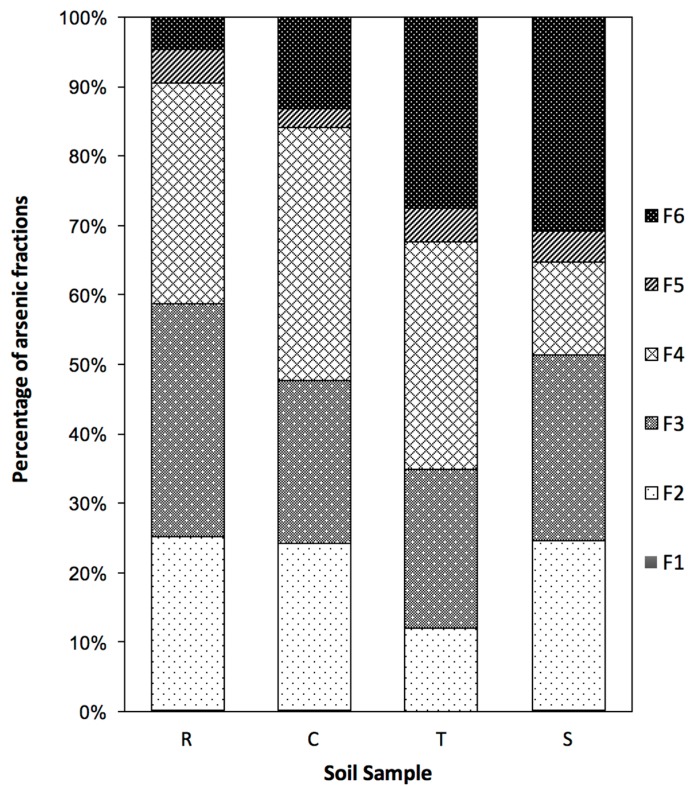
Speciation of arsenic using the modified sequential extraction method in four soil samples, including: R: River sediment, C: Mangrove forest soil, T: Paddy soil, S: Aquaculture sediment. (Fractions: F1: Non-specifically bound; F2: Specifically bound; F3: Amorphous Fe, Mn, Al oxides bound; F4: Crystalline iron bound; F5: Organic matter, secondary sulfide bound; F6: Residual).

The percentage of arsenic in six fractions of four soil samples was shown in Figure 1. In all samples, arsenic percentages in the non-specifically bound fraction (F1) were very small, with amounts lower than 0.2%. Thus, this fraction was not clearly displayed in Figure 1. In the second fraction (F2) that was extracted using (NH_4_)H_2_PO_4_ 0.05 M, the arsenic percentages were higher than in the F1 fraction with a range from 12% to 25%. The soil samples from the river bed, mangrove forest and aquaculture farm had similar percentages of the specifically bound fraction with an average value of 24.5%, while this fraction in the paddy soil sample was 12%.

The third fraction (F3) is As strongly bound to amorphous oxides of iron, manganese and aluminum. The amount of arsenic in the F3 fraction was 33%, 23%, 23% and 17% in the soil samples of the river bed, mangrove forest, paddy field and aquaculture farm, respectively. The first three fractions (F1, F2, F3) removed surface adsorbed arsenic from silicate clays and Fe, Mn, and Al oxides, which bound arsenic by outer and inner-sphere complexations. The remaining arsenic in the soil samples was co-precipitated with crystalline iron minerals and organic compounds.

In the fourth fraction (F4), arsenic co-precipitated with crystalline Fe in samples of the river bed, mangrove forest and paddy field, which had the highest arsenic content with percentages of 32%, 36%, and 33%, respectively. In contrast, the arsenic content obtained in the F4 fraction of the aquaculture farm soil samples occupied 14% of the total arsenic. The arsenic percentages in the F5 fraction of the four soil samples ranged from 3% to 5%, which showed that the organic matter and secondary sulfide bound As was low in all samples due to the low content of total organic carbon (from 0.7% to 1.5%). Finally, the arsenic content in the residual fraction (F6) in all samples ranged from 5% to 31% with an order of river bed < mangrove forest < paddy field < aquaculture farm.

### 3.3. Soil Washing Experiment Using Biodegradable Acids

#### 3.3.1. Arsenic Extraction

In previous studies, many authors obtained a significant amount of arsenic and iron extraction using ascorbic acid in a range from 0.01 to 0.1 M [47,48,49]. In this present study, 0.1 M ascorbic acid was adopted to combine with 0.2 M oxalic acid and 0.2 M citric acid to obtain the mixture solutions that were used in the washing experiment.

**Figure 2:**
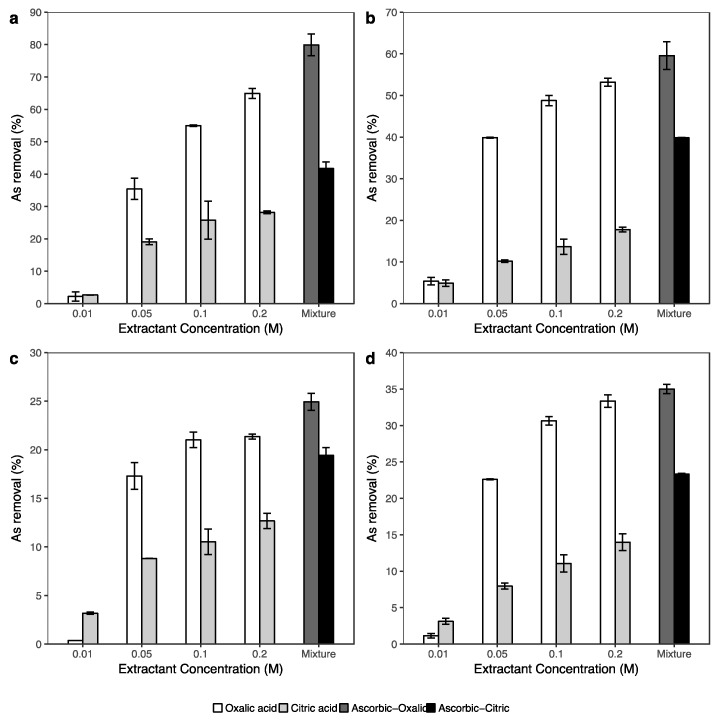
Effect of oxalic and citric acid in various concentrations, mixtures of oxalic (0.2 M) + ascorbic (0.1 M), and mixtures of citric (0.2 M) + ascorbic (0.1 M) on arsenic removal from river bed sediment (**a**), mangrove forest soil (**b**), paddy soil (**c**) and aquaculture farm sediment (**d**).

In this experiment, not only were there mixtures of oxalic-ascorbic acids, and citric-ascorbic acids, but various concentrations of single solutions of oxalic and citric acids were also used to determine the effects of arsenic removal. The results of the washing experiment are shown in Figure 2, where the results demonstrate that in a single extraction, the amount of arsenic removal simultaneously increased as the oxalic or citric acid concentration increased from 0.01 M to 0.1 M, while arsenic removal slightly increased when the single extractant solutions changed from 0.1 M to 0.2 M. The highest levels of arsenic removal in all samples were obtained using oxalic acid and citric acid at a concentration of 0.2 M.

In addition, the efficiencies of oxalic acid and citric acid were very different in all soil samples. In the single extraction using 0.2 M oxalic acid, the percentages of arsenic removal reached approximately 65%, 53%, 21% and 33% in river bed sediment, mangrove forest soil, paddy soil and aquaculture farm sediment, respectively. In contrast, the maximum amount of arsenic removal using 0.2 M citric acid was only 28% in river bed sediment. Additionally, only 13%, 14% and 18% of arsenic in paddy soil, aquaculture farm sediment, and mangrove forest soil samples, respectively, were removed using 0.2 M citric acid.

The addition of 0.1 M ascorbic to 0.2 M oxalic and 0.2 M citric acids enhanced arsenic extraction in all samples. In comparison with 0.2 M of the single extractants, the efficiencies of arsenic removal using mixtures of 0.1 M ascorbic and 0.2 M oxalic acids increased up to 80%, 60%, 25% and 35% in river bed sediment, mangrove forest soil, paddy soil, and aquaculture farm sediment samples, respectively. A similar trend also occurred under the synergistic effect of the ascorbic and citric acid mixture on arsenic removal in all samples. The efficiencies of the ascorbic and citric acid mixture on arsenic removal increased from 28% to 42% in river bed sediment, from 18% to 40% in mangrove forest soil, from 13% to 19% in paddy soil, and from 14% to 23% in aquaculture farm sediment in comparison to the extraction using 0.2 M citric acid.

#### 3.3.2. Iron, Aluminum and Manganese Extraction

**Figure 3:**
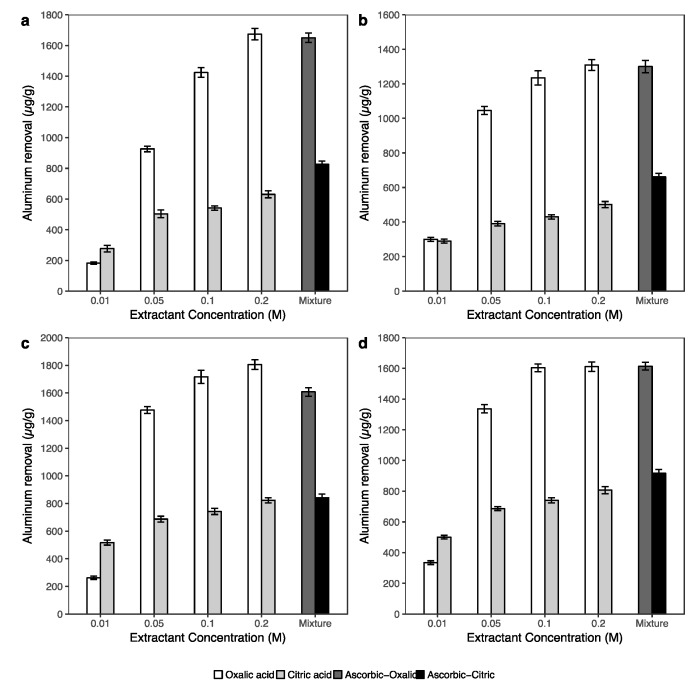
Extracted aluminum under the effect of oxalic and citric acid in various concentrations (from 0.01 M to 0.2 M), and the combination of 0.1 M ascorbic with 0.2 M oxalic, and the combination of 0.1 M ascorbic with 0.2 M citric acid in river bed sediment (**a**), mangrove forest soil (**b**), paddy soil (**c**) and aquaculture farm sediment (**d**).

**Figure 4:**
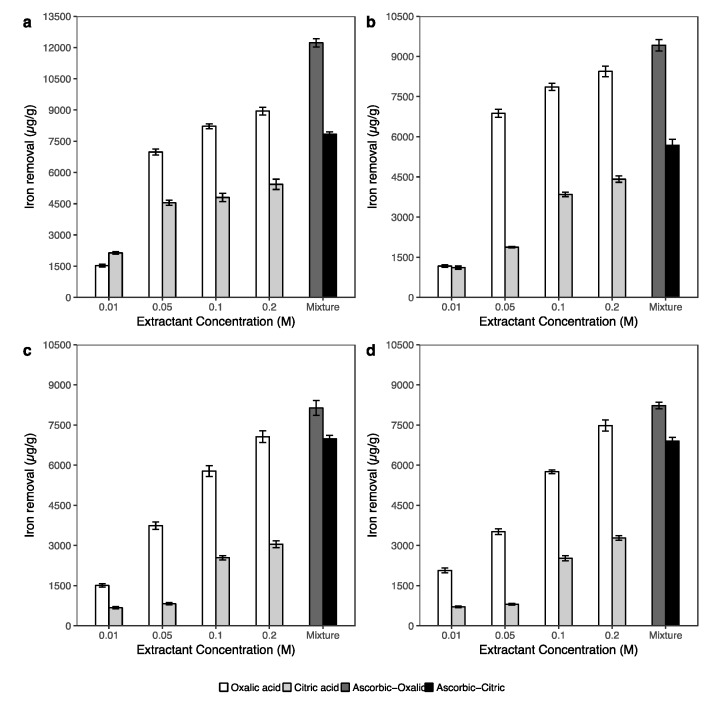
Extracted iron under the effect of oxalic and citric acid in various concentrations (from 0.01 M to 0.2 M), and the combination of 0.1 M ascorbic with 0.2 M oxalic acid, and the combination of 0.1 M ascorbic with 0.2 M citric acid in river bed sediment (**a**), mangrove forest soil (**b**), paddy soil (**c**) and aquaculture farm sediment (**d**).

To understand arsenic extraction via the dissolution of iron, aluminum and manganese in soil, the concentrations of these metals in the extracted solutions of the washing experiment were determined. Figure 3 and Figure 4 show, respectively, the concentration of extracted aluminum and iron under the effects of single solutions (oxalic and citric acid) and the combination solution of oxalic and citric acids with ascorbic acid.

The concentrations of Fe and Al in the extracted solutions under the effect of single extractant solutions had a similar trend, in which the amount of Fe and Al removal increased along with the concentration increasing for the oxalic and citric acid solutions. The results in Figure 3 and Figure 4 show the high removal efficiency of oxalic acid on not only iron but also aluminum in soil. The amount of Al in the extracted solution sharply increased when the concentration of oxalic acid increased from 0.01 to 0.2 M. In contrast, the efficiency of citric acid in Al removal was approximately two times lower than those in oxalic acid extractions. The addition of 0.1 M ascorbic acid did not have a significant effect on Al extraction in both the combinations with 0.2 M oxalic and 0.2 M citric acid.

The highest concentrations of iron removal were in the river bed sediment sample (Figure 4a), with approximately 12,200 μg g^−1^ that was extracted using the combination of ascorbic acid and oxalic acid. Under the same extractant conditions, the amount of iron released from the mangrove forest soil (Figure 4b) was 9400 μg g^−1^, while the extracted iron concentrations in the paddy soil (Figure 4c) and aquaculture farm sediment (Figure 4d) were 8100 μg g^−1^ and 8200 μg g^−1^, respectively. In all soil samples, ascorbic significantly affected iron dissolution, especially in the samples of paddy soil and aquaculture sediment. In the paddy soil, Fe concentration increased from approximately 3000 μg g^−1^ in the 0.2 M citric acid extraction to 7000 μg g^−1^ in the ascorbic and citric combination solution, while in the aquaculture sediment sample, this value increased from 3300 μg g^−1^ to approximately 7000 μg g^−1^. The trend of iron extraction was similar to the arsenic release that was shown in Figure 2.

### 3.4. Arsenic Fraction after the Washing Experiment

**Figure 5:**
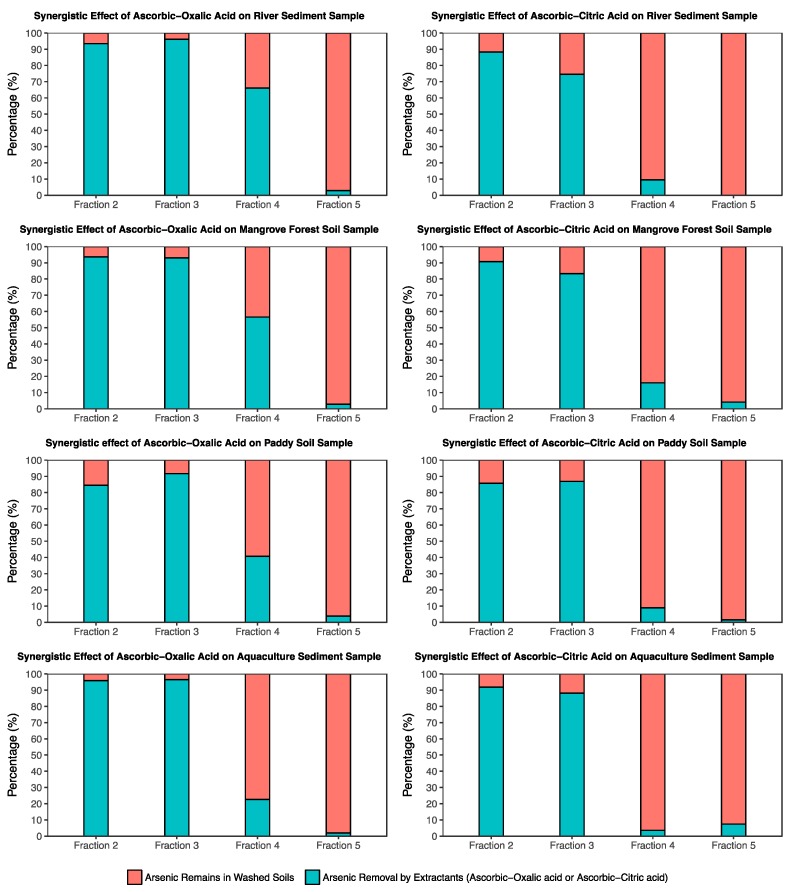
Percentages of arsenic removal and remnants in soils under the synergistic effect of ascorbic acid with each single solution of oxalic and citric acids.

To determine the effectiveness of extractant solutions on the main arsenic fractions, soil samples were first washed using an ascorbic-oxalic mixture and an ascorbic-citric mixture and then extracted using sequential extraction methods. Base on the contents of arsenic fractions in initial samples and washed soils, the percentages of arsenic that were eliminated by extractants (ascorbic-oxalic acid, and ascorbic-citric acid) were calculated. The percentages of arsenic remained in washed soils (red colour) and arsenic removal by extractants (green colour) in the four fractions (F2, F3, F4 and F5) is shown in Figure 5.

Obviously, complete removal of arsenic was not achieved after the washing procedure under the synergistic effect of either citric and ascorbic acids, or oxalic and ascorbic acids. These extractant solutions had a significant effect on removing arsenic in specifically bound (F2) and amorphous Fe, Mn, Al oxides bound (F3). Nevertheless, the efficiencies of arsenic removal using these extractants decreased in fractions of crystalline iron oxide-hydroxide bound (F4), and only small amounts of arsenic were removed in organic and secondary sulfide bound fractions (F5).

In all samples, the effectiveness of arsenic removal using the combination of ascorbic-oxalic acid was higher than that of the ascorbic-citric acid combination, especially in arsenic removal from the crystalline oxide-hydroxide of iron. In the river bed sediment sample, 66% of the arsenic was removed using the ascorbic-oxalic acid combination, while only 9.5% of the arsenic was washed using the ascorbic-citric acid combination. Similarly, the arsenic amount in the samples of mangrove forest soil, paddy soil, and aquaculture farm sediment that was removed using the ascorbic- oxalic acid combination was 57%, 41% and 23%, respectively. In contrast, arsenic removal using the ascorbic-citric acid combination in the same samples was 16%, 9% and 4%, respectively.

## 4. Discussion

### 4.1. Toxicity Potential of Arsenic in Soil Samples

Arsenic is a ubiquitous trace metalloid; however, concentrations of As in non-contaminated soils are typically well below 10 μg g^−1^ [50]. In this study, all soil samples were contaminated by arsenic with a range of As concentrations from 16.84 to 31.0 μg g^−1^ (Table 2). In addition, these samples primarily existed in the flooded environment where reducing conditions are dominant. According to Patrick [51], under moderately reduced soil conditions (0–100 mV), arsenic solubility was controlled by the dissolution of iron and manganese (oxyhydr)oxides, and upon reduction to −200 mV, the solubility of As continuously increased 13-fold compared to 500 mV. The activity of As in the soil solution is most commonly by surface complexation reactions on oxides/hydroxides of Al, Mn and Fe [50,52]. In the four investigated soil samples, arsenic contents were high in not only the exchangeable fraction (F2) but also the amorphous bound fraction of Fe, Mn and Al (Figure 1). Under anaerobic conditions like river bed, aquaculture pond, mangrove forest (wetland), or paddy field, iron hydroxides readily dissolve, and As is released into the soil solution, where As would be present primarily as As(III) [53]. In addition, arsenic can also be released from As-bearing crystalline iron via microorganism activity under reducing conditions even though this process is very slow [54].

The arsenic fractions in the four soil samples indicated that the potential of As release in the paddy soil sample was lower than the other samples due to low concentrations of total arsenic, and the arsenic content in this sample was dominantly in the As-bearing crystalline iron fraction (F4). In contrast, the river bed sediment, which contained approximately 60% As in exchangeable and Fe, Mn and Al amorphous fractions, has a high potential for As release into the environment.

### 4.2. Effectiveness of Biodegradable Organic Acid in Arsenic Removal

The results of arsenic extraction in the washing experiment indicated that biodegradable acids, such as oxalic, citric and its combination with ascorbic, can be considered as a means of remediating As-contaminated soils. This is consistent with the outcome of other authors [31,47,55].

In previous studies, the efficiency and mechanisms of organic acid on metal extraction were reported [33,47,56]. The primary chemical dissolution mechanisms of metals using these organic acids are predominantly through acidification, complexation and reduction [57,58]. Depending on their dissolution constants and number of carboxylic groups, organic acid may carry a varying negative charge [32]. The organic acids that contain only one carboxyl group have a limited metal complexing ability, while citric, malic, and oxalic acids and their salts have a high affinity for metal ions such as Fe^3+^, Al^3+^, and Mn^2+^ [32,59].

The efficiency of oxalic and citric acid mobilizes arsenic from soils and sediments by effectively dissolving As-bearing Fe (oxyhydr)oxides via a ligand-promoted reaction with two steps [33,47,56]. The first step of the dissolution reaction is the adsorption of carboxylic acid on the surface of iron (oxyhydr)oxides and then formation of the surface complexation on the system interface; the second step is the detachment of the iron complex to the aqueous environment, as follows:

≡Fe^III^–OH^+^ + L^n−^ + H^+^ → [≡Fe^III^–L]^−(n−2)^ + H_2_O (1)

[≡Fe^III^–L]^−(n−2)^ + H^+^ → [Fe^3+^ –L]^−(n−3)^_(aq)_ + H≡ (2)

where L^n−^ is the organic ligand with oxidation number n; (aq): aqueous.

According to Panias et al. [60], under acidic conditions, organic acids such as oxalic and ascorbic, can involve the generation of Fe^2+^ ions in the solution via the reduction process:

[≡Fe^III^–L]^−(n−2)^ ↔ [≡Fe^II^–L^−(n−2)^]^−(n−2)^ (3)

[≡Fe^II^–L^−(n−2)^]^−(n−2)^ → Fe^2+^_(aq)_ + products of ligand oxidation (4)

Fe^2+^_(aq)_ + L^n−^ → [Fe^2+^ –L]^−(n−2)^_(aq)_ (5)

Based on these reactions, the phenomenon of Al, Fe and Mn dissolution in Figure 3, Figure 4 and Figure S2 could be interpreted. In this study, not only iron but also aluminum and manganese were significantly extracted using 0.01 M oxalic and 0.1 M citric acids with an initial pH of 3.5. Under acidic conditions, the (oxyhydr)oxides of Fe, Al and Mn on soil surfaces easily reacted with oxalic and citric acid via ligand-promoted reaction and formed surface complexes and aqueous complexes as in reactions (1) and (2).

In general, the low pH condition can potentially lead to the dissolution of some As-bearing minerals and also direct desorption of As [33,61]. However, the amounts of As (Figure 2) and Fe and Al (Figure 3 and Figure 4) extracted by oxalic acid were significantly higher than those by citric acid. These results indicated that the enhanced arsenic extraction using oxalic acid depends on not only the lower pH but also on the dissolution of As-bearing Fe, Al and Mn (oxyhydr)oxide in soils of oxalic acid. The low extraction efficiencies of citric acid for Fe and Al in comparison with oxalic acid may be caused by the stability constants of this acid with Fe and Al in the complexation reaction. According to Martell and Smith [62], the stability constants (logK) of citric acid for the formation of Fe^3+^-citrate and Al^3+^-citrate from Fe^III^ and Al^III^ are 11.2 and 7.98, respectively, while these values of oxalic acid are 18.6 and 14.88, respectively. Furthermore, oxalate is a mild reducing agent and is able to promote the dissolution of iron via reduction reaction to form ferrous oxalate [63]. When a sufficient number of ferrous oxalate ions have been formed, the secondary reductive dissolution step becomes operative, thus increasing the iron dissolution efficiency of the whole process [60].

In Figure 2 and Figure 4, the significant increase in extracted As and Fe using the ascorbic and citric acid combination indicated that ascorbic acid had an important role on iron dissolution and thus assisting in arsenic removal. According to Dos Santos Afonso [64], ascorbic acid and its salts are strong reducing agents. The ability of ascorbate (C_6_H_7_O_6_^2−^) on the reductive dissolution of iron was suggested to occur via outer-sphere electron transfer of the acidified ascorbate [65,66]. In the research of synergistic effects of the combination of oxalate and ascorbate, Lee et al. [48] reported that in the same condition, most of the extracted iron by oxalate was in the Fe^3+^ form, while in ascorbate extraction, the Fe^2+^ form was dominant. In addition, the amount of Al release (Figure 3) was not change much between the extractions of single acids (0.2 M oxalic acid or 0.2 M citric acid), and the combination of ascorbic-oxalic or ascorbic-citric acid. Therefore, this evidence highly supports that the combination of ascorbic and single acid solutions significantly affected arsenic via the dissolution of iron in soil samples. The relationships between extracted arsenic and extracted iron, aluminum, manganese in Figure S3 also supports this statement.

Comparing the release of arsenic from the four fractions among all samples (Figure 5), particularly the arsenic in the iron crystalline fraction (F4), demonstrates the high efficiency of the ascorbic and oxalic combination on arsenic removal as well as the effectiveness of these extractants on soil fractions. Figure 5 shows that almost all the arsenic in fraction 2 (exchangeable) and fraction 3 (Fe, Al and Mn amorphous bound) in all samples were removed using both ascorbic-oxalic and ascorbic-citric acid. However, the significant amount of arsenic in fraction 4 (crystalline iron) was only found in the extraction of the oxalic and ascorbic acid combination. In contrast, under extraction using the ascorbic and citric acid combination, only a small amount of As was removed. These results indicated that both of the combination extractants have high effectiveness on the removal of arsenic that is bound with amorphous iron, aluminum and manganese, but the oxalic and ascorbic combination showed higher efficiency in dissolving As-bearing crystalline iron.

## 5. Conclusions

In this study, arsenic extraction from four soil samples was investigated using single acids, including oxalic and citric, and the combination of these acids with ascorbic acid. In addition, detailed information of the arsenic fractions in all soil samples before and after treatment using the extractants was also determined using a modified method. According to the results obtained from current study, the following conclusions were derived as follows:

(1) Most of the arsenic in the four investigated soils was bound with amorphous Fe, Mn (F3), and crystalline Al and iron (F4) and was in the exchangeable fraction (F2). The As content in the organic matter fraction (F5) ranged from 3–5% in all samples.
(2) Biodegradable organic acids showed high potential for remediation the arsenic contaminated soil. However, the efficiency of As removal using the combination of 0.1 M ascorbic with 0.2 M oxalic and/or 0.2 M citric acids was better than the single acid (0.2 M oxalic and/or 0.2 M citric acid) extractions.
(3) The results of extracted arsenic using the combination of 0.2 M oxalic acid and 0.1 M ascorbic acid solution suggested that this solution can effectively wash arsenic from soils associated with crystalline Fe oxides. In addition, the solution of 0.2 M citric and 0.1 M ascorbic acids can be applied to arsenic-contaminated soil, which has a low crystalline Fe oxide content in soil.
(4) The amount of extracted arsenic under the effects of biodegradable acids was strongly correlated with the dissolution of iron and aluminum in soils.
(5) The soils treated using biodegradable acids had low remaining concentrations of arsenic that are primarily contained in crystalline iron oxides and organic matter fractions.

## Figures and Tables

**Table 1:** Modified sequential extraction scheme for arsenic in soils used in this study.

Fraction	Reagents	Extraction Condition	Arsenic Form	Reference
F1	0.05 M (NH_4_)_2_SO_4_	1:25 of soil to solution. 4-h shaking, 20 °C	Non-specifically bound	[23,24,26]
F2	0.05 M (NH_4_)H_2_PO_4_	1:25 of soil to solution. 16-h shaking, 20 °C	Specifically bound	[23,24,26]
F3	0.2 M NH_4_-Oxalate, pH = 3.25	1:25 of soil to solution. 6-h shaking in the dark, 20 °C	Amorphous iron, aluminum, manganese oxide bound	[23,24,26]
F4	0.2 M NH_4_-Oxalate + 0.1 M Ascorbic acid; pH = 3.25	1:25 of soil to solution. 30 min occasional agitation, 96 °C	Crystalline iron	[23,24,26]
F5	H_2_O_2_ + HNO_3_ acid, pH = 2, 3.2 M NH_4_OAc in 20% HNO_3_	3-h occasional agitation at 85 °C	Organic matter and secondary sulfide bound	[37,38,39]
Total As	HNO_3_ acid, H_2_O_2_	Hot-plate, 110 °C	Total concentration of arsenic	[40]
F6	Total As—(F1 + F2 + F3 + F4 + F5)		Residual	

**Table 2:** The mean values of some major soil properties for four soil samples used in the experiments.

Sample	Land Uses	Latitude	Longitude	pH	OC	CEC	Clay Content	Silt Content	Sand Content	Astot
					(%)	(cmol kg-1)	(%)			(µg/g)
R	River bed	20.34	106.39	6.92	1.03	13.2	5.5	49.3	45.2	31.0
C	Mangrove forest Soil	20.25	106.57	6.98	0.70	11.0	18.1	47.8	34.1	27.4
T	Paddy soil	20.27	106.45	6.88	1.50	11.4	25.3	58.1	16.6	16.8
S	Aquaculture farm	20.25	106.55	6.52	1.20	16.0	26.2	44.4	29.4	17.5

OC: Organic carbon content; CEC: Cation exchange capacity. As_tot_: Total concentration of arsenic.

## Supplementary Materials

The following are available online at www.mdpi.com/1660-4601/14/9/990/s1, Figure S1: Study area and sample sites in Ba-Lat estuary, Vietnam (R: River sediment, C: Mangrove forest soil, T: Paddy soil, S: Aquaculture sediment); Figure S2: Extracted manganese under the effect of oxalic and citric acid in various concentrations (from 0.1 M to 0.2 M), and the combination of 0.1 M ascorbic with 0.2 M oxalic acid, and the combination of 0.1 M ascorbic with 0.2 M citric acid in river bed sediment (a), mangrove forest soil (b), paddy soil (c) and aquaculture farm sediment (d); Figure S3: The relationship between extracted arsenic and extracted iron, aluminum and manganese under the effect of oxalic, citric and combination of these acids with ascorbic acid in four soil samples.
