# Supplementary material for: Arsenic Speciation and Extraction and the Significance of Biodegradable Acid on Arsenic Removal—An Approach for Remediation of Arsenic-Contaminated Soil
Source: Int J Environ Res Public Health. 2017 Aug 31;14(9):990. doi: 10.3390/ijerph14090990 (PMC5615527; doi:10.3390/ijerph14090990)
Supplement: Supplementary file 1 [file ijerph-14-00990-s001.pdf]

Supplementary Data

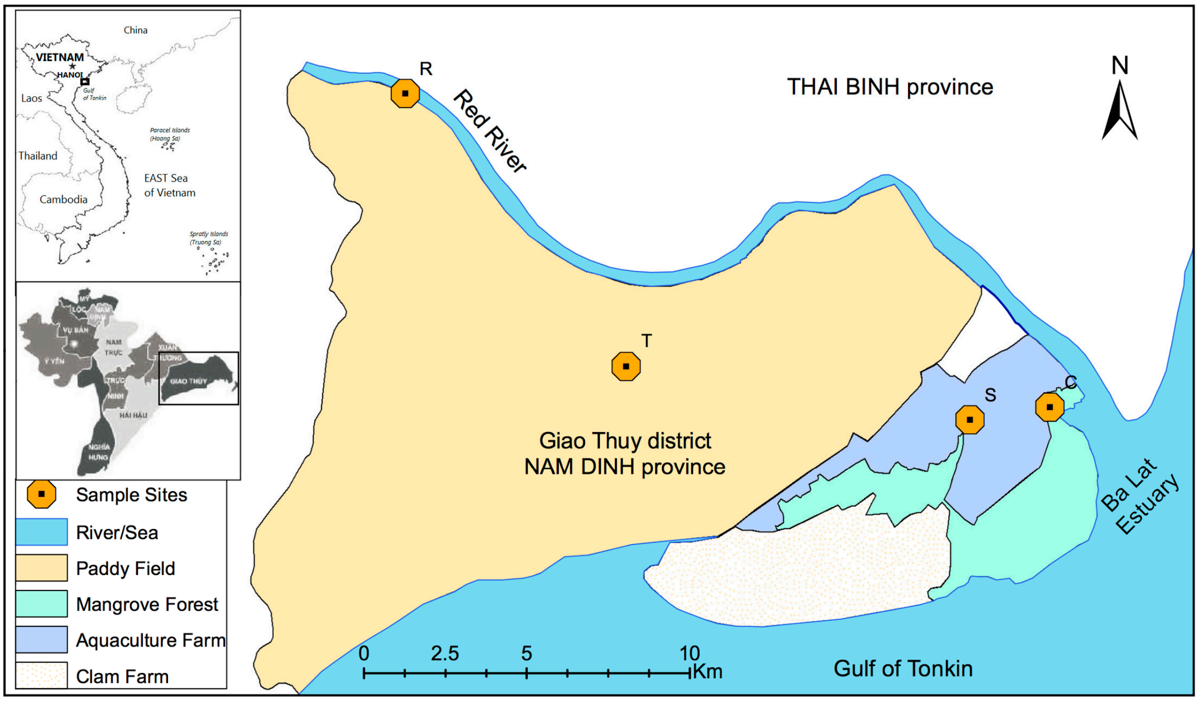

Figure S1. Study area and sample sites in Ba-Lat estuary, Vietnam (R: River sediment, C: Mangrove forest soil, T: Paddy soil, S: Aquaculture sediment).

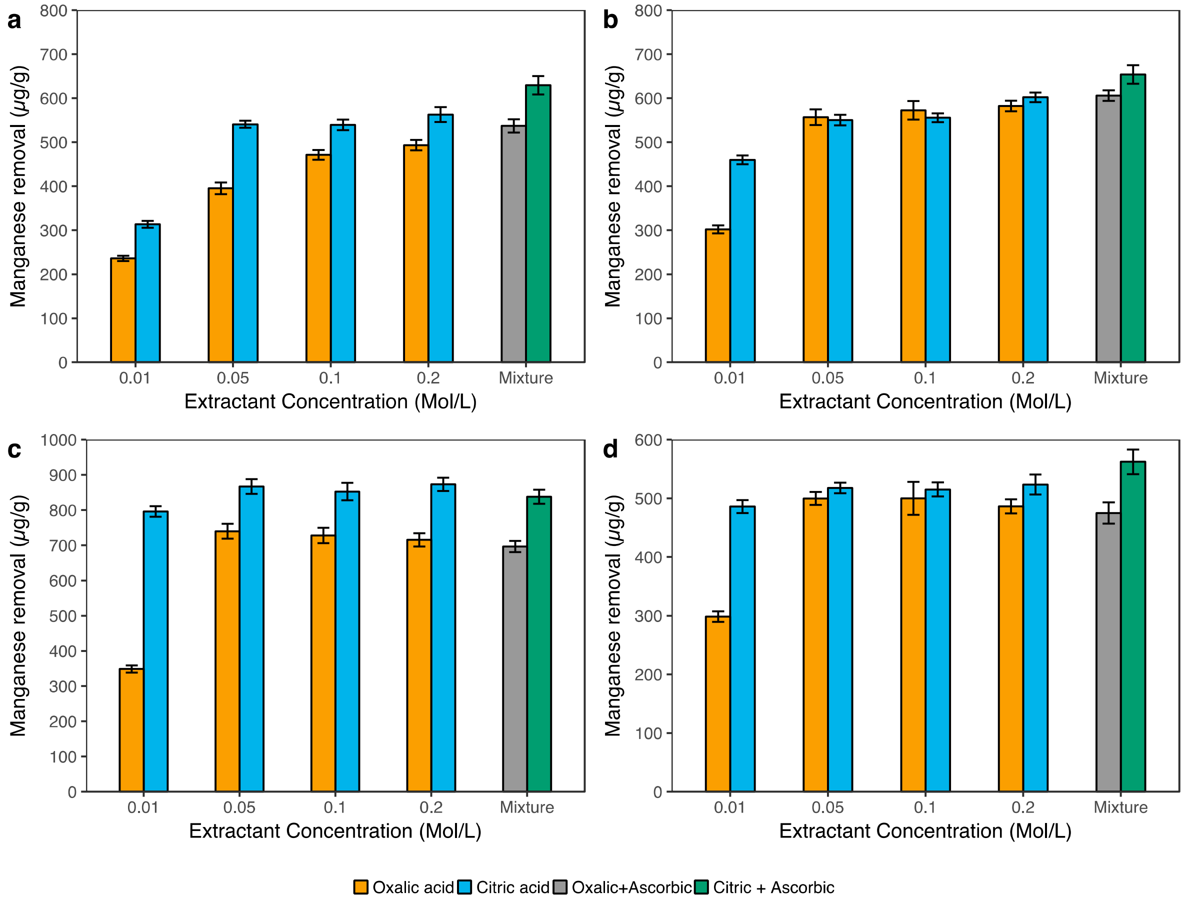

Figure S2. Extracted manganese under the effect of oxalic and citric acid in various concentrations (from 0.1 M to 0.2 M), and the combination of 0.1 M ascorbic with 0.2 M oxalic acid, and the combination of 0.1 M ascorbic with 0.2 M citric acid in river bed sediment (a), mangrove forest soil (b), paddy soil (c) and aquaculture farm sediment (d).

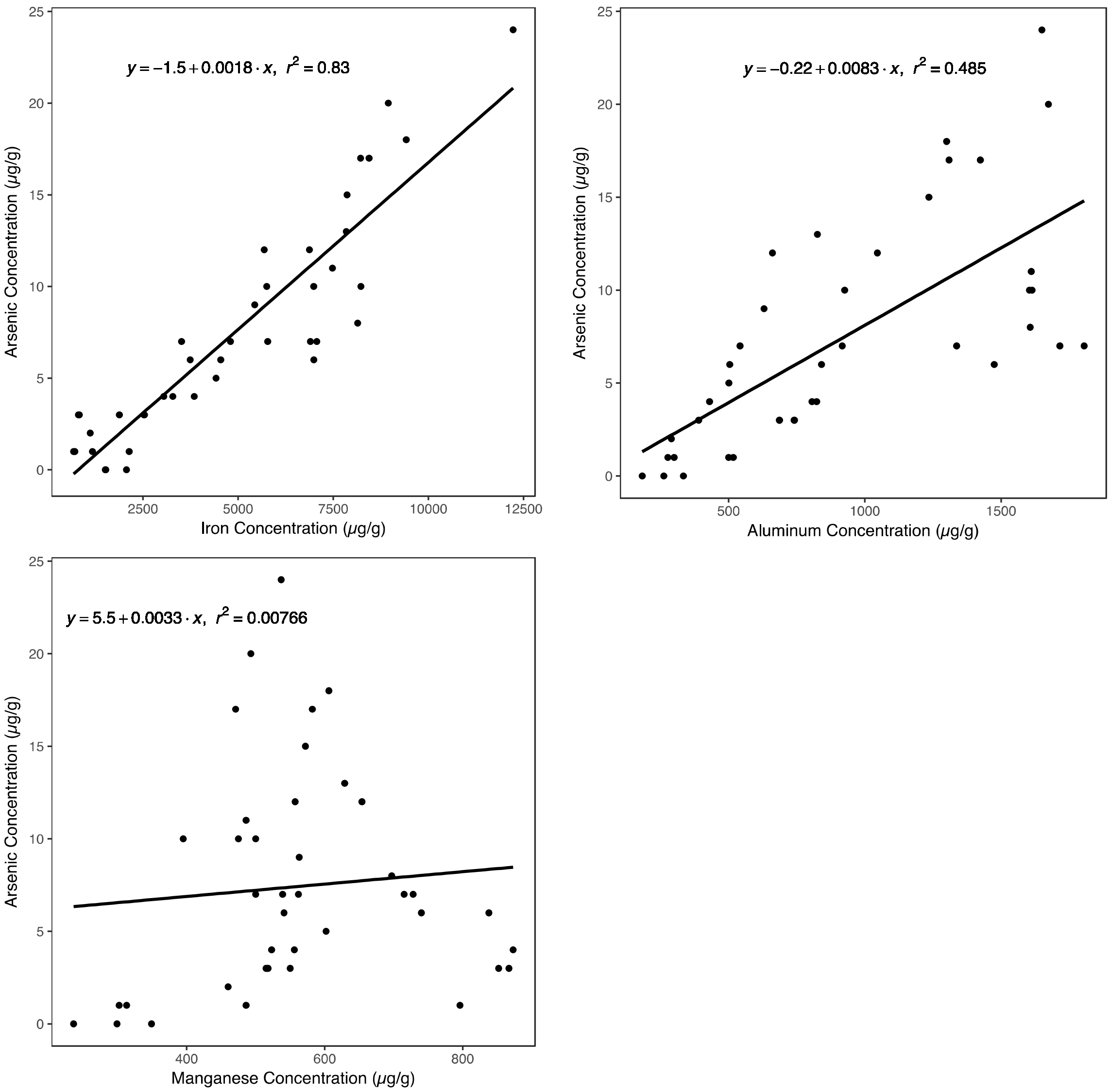

Figure S3. The relationship between extracted arsenic and extracted iron, aluminum and manganese under the effect of oxalic, citric and combination of these acids with ascorbic acid in four soil samples.
